# Evidence of an Own-Age Bias in Facial Emotion Recognition for Adolescents With and Without Autism Spectrum Disorder

**DOI:** 10.3389/fpsyt.2020.00428

**Published:** 2020-06-03

**Authors:** Kathryn M. Hauschild, Peter Felsman, Cara M. Keifer, Matthew D. Lerner

**Affiliations:** ^1^ Social Competence and Treatment Laboratory, Department of Psychology, Stony Brook University, Stony Brook, NY, United States; ^2^ Alan Alda Center for Communicating Science, Stony Brook University, Stony Brook, NY, United States; ^3^ Department of Psychology, University of Virginia, Charlottesville, VA, United States

**Keywords:** face processing, perceptual expertise, own-age bias, emotion recognition, autism spectrum disorder, adolescents

## Abstract

A common interpretation of the face-processing deficits associated with autism spectrum disorder (ASD) is that they arise from a failure to develop normative levels of perceptual expertise. One indicator of perceptual expertise for faces is the own-age bias, operationalized as a processing advantage for faces of one's own age, presumably due to more frequent contact and experience. This effect is especially evident in domains of face recognition memory but less commonly investigated in social-emotional expertise (e.g., facial emotion recognition; FER), where individuals with ASD have shown consistent deficits. In the present study, we investigated whether a FER task would elicit an own-age bias for individuals with and without ASD and explored how the magnitude of an own-age bias may differ as a function of ASD status and symptoms. Ninety-two adolescents (63 male) between the ages of 11 and 14 years completed the child- and adult-face subtests of a standardized FER task. Overall FER accuracy was found to differ by ASD severity, reflecting poorer performance for those with increased symptoms. Results also indicated that an own-age bias was evident, reflecting greater FER performance for child compared to adult faces, for all adolescents regardless of ASD status or symptoms. However, the strength of the observed own-age bias did not differ by ASD status or severity. Findings suggest that face processing abilities of adolescents with ASD may be influenced by experience with specific categories of stimuli, similar to their typically developing peers.

## Introduction

Individuals with autism spectrum disorder (ASD) demonstrate impaired categorization of the emotional facial expressions of others (1–3). Some have hypothesized that this characteristic deficit may arise from a failure to develop normative levels of face-relevant perceptual expertise (4–6). Face-relevant expertise develops with frequent and recent experience (7–9), resulting in improved performance on behavioral tasks of recognition memory and emotion identification for faces of one's own age compared to faces of another age, known as the own-age bias [OAB; (10–12)]. Thus, if ASD-related deficits in FER can be explained by a failure to develop normative levels of face-relevant perceptual expertise, then people with ASD may not be expected to demonstrate an OAB to the same extent as their peers. However, whether such a bias occurs among individuals with ASD to the same extent as their peers has not been tested. The current study addresses this question.

Although the magnitude and universality of emotion perception deficits associated with ASD have been debated [see (1, 13) for review], recent meta-analyses support the presence of behavioral deficits in facial emotion recognition (2, 3). Early accounts of these deficits posited that observed group differences in performance may have been driven by difficulty in processing negative (14) or threat-relevant (i.e. fearful) emotions (15, 16). However, more recent work indicates that individuals with ASD perform worse than typically developing individuals on tasks of facial emotion identification and recognition across all six basic emotions (2, 17). These deficits appear to be particularly pronounced during the completion of tasks that require judgments related to more subtle expressions of emotion [(5, 18); but also see (19)]. Additionally, eye tracking, electrophysiological, and neuroimaging data suggest atypical attentional and cognitive processing of emotional faces by individuals with ASD (20–22).

One explanation of FER deficits in ASD is that those with ASD fail to develop normative levels of face-relevant perceptual expertise (4, 6). For typically developing individuals, increased experience with categories of face stimuli, such as human faces compared to macaque faces (23), confers an expert level processing advantage for stimuli of that category (8, 24). This advantage is related to the ability to integrate previously experienced exemplars into prototypic mental representations (9, 25) and engage in configural processing (26). Individuals with ASD, on the other hand, have failed to demonstrate many of these same markers (27, 28). Moreover, developmental studies of facial emotion recognition have noted that deficits in FER for individuals with ASD increase with age, observing the greatest divergence between group performance trajectories in adulthood (5, 29, 30), when typically developing individuals demonstrate expert performance levels. Together these patterns of findings suggest that face-relevant perceptual representations of individuals with ASD may not be as sensitive to experience as those of their typically developing peers.

The idea that face-relevant perceptual experience leads to expertise is demonstrated in the own-age bias (OAB). The OAB constitutes a processing advantage for own- compared to other-aged faces that is contingent upon greater contact with individuals of one's own age (11, 12). This bias is dependent upon differential experience with face age (31, 32), is reduced by visual exposure training of other-aged faces (33), and has been reliably observed in both tasks of face recognition memory (11) and FER (10, 34). As is true of many indicators of perceptual expertise, the OAB is also reflected in differential patterns of visual attention to own-age faces (35–39) and the recruitment of specialized cortical networks when processing own-age faces (36, 40).

The OAB likely reflects the influence of increased contact and visual experience with faces of one's age cohort (11; for an alternate account see 41); thus, signaling ongoing plasticity in perceptual representations as they adapt to the changing facial structures of peers over time. This is in contrast to a processing advantage for adult faces regardless of one's own current age that would be predicted if age biases in FER were due to cumulative lifetime visual experience. Exceptions to OAB in the literature support this account; for example, infants who spend most of their time with adults show a processing advantage for adult faces (42) and teachers working with young children are equally accurate in identifying child and adult faces (33).

The same principle underlying the OAB is demonstrated in the other-race effect (ORE). The ORE constitutes a processing advantage for own- compared to other-race faces that is contingent upon greater contact with individuals of one's own race (see 43 for review). However, unlike the OAB, the ORE likely reflects a summation of visual experience across development, not merely the most recently encountered exemplars (43). Findings from the ORE literature suggest that individuals with ASD may indeed be sensitive to cumulative visual experience with faces, reflected in greater performance accuracy for own- compared to other-race in tasks of facial recognition memory (44, 45). Although, this has not always been replicated (46, 47). Furthermore, it remains unknown whether such effects extend to tasks of FER, where individuals with ASD demonstrate increased impairment (1).

To date, no work has examined whether the strength or direction of the OAB varies for individuals with ASD. In order to better understand the role of experience in face processing for individuals with ASD, the present study aimed to explore whether adolescents with and without ASD would evidence an OAB while completing a task of FER; that is, demonstrate greater performance accuracies in emotion identification for own-age compared to other-age faces. Given the well-documented deficits in FER (1–3), we predicted that individuals with ASD would demonstrate poorer FER accuracy compared to controls regardless of the stimulus face age or emotion. We also predicted that individuals with ASD would demonstrate greater performance deficits across all four emotions compared to their typically developing peers, regardless of stimulus face age. Based on previous findings (11), it was predicted that adolescents without ASD would demonstrate an own-age bias. Due to conflicting evidence on whether individuals with ASD are as sensitive to visual experience with faces (27, 44, 45, 47), analyses related to how the magnitude of any observed OAB would be attenuated by ASD status and severity or differ by emotion were considered exploratory in order to support future hypothesis generation; thus, no specific predictions were made at this time.

Previous quantitative work has found that social deficits associated with ASD are not clearly diagnostically differentiable and may thus be best modeled continuously rather than categorically (48–50). This effect is well illustrated in work examining the broader autism phenotype, an occurrence of sub-clinical ASD-like traits often observed in the genetic relatives of individuals with ASD (51). Therefore, the relationships between FER and OAB were also tested against a dimensional measure of ASD symptom severity, the ADOS-2 comparison score (ADOS-2 CS). It was hypothesized that if an overall deficit in FER or magnitude of any observed OAB did not differ by diagnostic group, these measures may vary by symptom severity assessed across the full sample.

## Materials and Methods

### Participants

One hundred adolescents between the ages of 11 and 14 years were recruited from the greater Long Island area (**Table 1**). Eight individuals (non-ASD *n* = 1; ASD *n* = 7) were ineligible following initial screening due to a full-scale intelligence quotient (FSIQ) <70 (presence of intellectual disability) as determined by the Kaufman Brief Intelligence Test-Second Edition (KBIT-2; 52). The remaining 92 eligible participants were assessed and classified into one of two groups: ASD (*n* = 52, 38 male) or non-ASD (*n* = 40, 25 male), using cutoffs determined by the Autism Diagnostic Observation Schedule-Second Edition (ADOS-2; 53) administered by research-reliable examiners. ADOS-2 Comparison Scores (CS) were computed for all participants as a dimensional measure of ASD symptom severity (54). Groups did not differ by age (*t*(90) =.90, *p* =.37) or IQ (*t*(90) =.29, *p* =.77). Informed consent was obtained from the guardians of all study participants and all participants assented to study procedures prior to participation. All study procedures were approved by the Institutional Review Board of Stony Brook University and conform to Common Rule standards.

**Table 1:** Descriptive Statistics and Independent Samples t-Tests Comparing Age, Full-Scale IQ, and ADOS-2 CS Across Participants in the ASD and Non-ASD Groups.

	ASD (N = 52, 38 male)	Non-ASD (N = 40, 25 male)	*t*	df
Racial or Ethnic Minority (*n*, %)	7, 13.5%	10, 25%	–	–
Parental Education (*n*, %) High School Degree Some College College Graduate Graduate or Professional Degree	3, 5.8% 15, 28.8% 21, 40.4% 13, 25%	3, 7.5% 10, 25% 14, 35% 13, 32.5%	–	–
Yearly Household Income (*n*, %) Less than $30,000 $30,000–$75,000 $75,000–$120,000 $120,000–$165,000 Greater than $165,000 Declined to Answer	4, 7.7% 6, 11.5% 19, 36.5% 5, 9.6% 14, 26.9% 4, 7.7%	1, 2.5% 7, 17.5% 17, 42.5% 5, 12.5% 8, 20.0% 2, 5.0%	–	–
Age (*M*, *SD)*	12.64, 1.10	12.85, 1.07	.90	90
Full-Scale IQ (*M*, *SD)*	105.67, 14.08	106.55, 14.79	.29	90
ADOS-2 CS (*M*, *SD)*	7.58, 2.05	1.83,.87	−16.59***	90

***p <.001. ADOS-2 CS, Autism Diagnostic Observation Schedule–Second Edition Comparison Score.

### Facial Emotion Recognition Task

**Figure 1:**
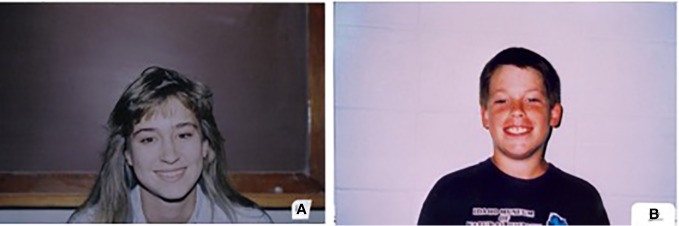
Example images from the Diagnostic Analysis of Nonverbal Accuracy-2 depicting happy expressions from the **(A)** adult face subtest and **(B)** child face subtest.

Participants completed a computerized version of the adult and child facial expression subtests from the Diagnostic Analyses of Nonverbal Accuracy 2 (DANVA-2; 55), a standardized measure of facial emotion recognition that has been previously validated in typically developing and ASD samples (55, 56). Stimuli included 48 naturalistic color photographs of males (24) and females (24), depicting one of four emotions (12 happy, 12 sad, 12 angry, and 12 fearful, **Figure 1**). Within each subtest, presentation order of the four emotions is randomized; however, all participants viewed the photographs in the same, standardized order. All participants completed the task in a blocked fashion: adult face subtest, followed by the child face subtest. Images included in the adult subtest (24) were of individuals above the age of 18 years while images in the child subtest (24) were of individuals between the ages of 6 and 12 years. All faces were displayed in a frontal view and included the neck and torso of the individual photographed in front of either a chalkboard (the adult subtest) or a white brick wall (the child subtest).

During the task, participants were asked to view each face and make a behavioral determination of the emotion displayed (happy, sad, angry, or fearful) *via* button press. Stimulus presentation time varied from 1 to 3 s with trial advancement dependent upon participant response. In the event that a participant did not provide a response within the maximum 3 s time window, the face was removed from the screen, but the response options remained requiring the selection of a response to advance to the next trial.

### Data Analytic Plan

Percent accuracy of emotion recognition for adult and child face stimuli was calculated by dividing the total number of correct responses by the total number of stimuli (24) in the subtest. In order to ensure that all participant groups were able to perform above chance, one-sample t-tests were then carried out comparing the percent accuracy of FER for adult and child faces to the chance value of 25% (1/4 possible responses) separately for both the Non-ASD and ASD groups.

To test for an OAB and any attenuation by emotion or ASD status, a 2 (face age: adult, child) × 4 (emotion: happy, sad, angry, fearful) × 2 (diagnostic group: Non-ASD, ASD) mixed-design ANOVA was calculated. Pearson's correlations were used to test for a relationship between a measure of ASD symptom severity, ADOS-2 CS, and FER accuracy across the entire sample as well as in each diagnostic group separately. Following the proposed procedures of Edwards (1994; 57), a moderated regression model was used to probe the relationship between the magnitude of the own-age bias and ASD symptom severity in each group separately and then combined. All findings with *p* <.05 were considered to be statistically significant while findings of *p* =.05 to *p* =.08 were considered marginal and reported as such.

*Post-hoc* power analyses were conducted using G*Power 3.1 (58, 59) to determine whether the sample size recruited was sufficient to detect main effects of diagnostic group, face age, and emotion as well as the potential two-way interactions between the three primary variables of interest. Given the expected effect size (*d* =.40) for the main effect of diagnostic status on FER task accuracy reported by Uljarevic and Hamilton (3), an alpha of.05, and sample of 92 participants, the power for main effects was at least 0.66. Assuming an equivalent medium effect size of $\eta_{p}^{2}=.06,$ an alpha of .05, and sample of 92 participants, the power to detect interaction effects was at least .99. Analyses pertaining to the three-way interaction between diagnostic groups, stimulus age, and emotion were exploratory; therefore, power analyses could not be carried out.

Shapiro-Wilk's tests of normality indicated that our primary dependent variables of interest were not normally distributed. Therefore, we ran all follow-up paired comparisons bootstrapped as well as re-tested any significant differences nonparametrically. Additionally, as the distributions of our primary variables of interest were found to be skewed, we removed outliers more than two standard deviations outside of the mean performance accuracy for either the adult or child subtest (*n* = 4) and re-ran all analyses with no substantial changes in findings. Results from these additionally analyses were consistent with our primary findings; therefore, in order to best represent the FER ability observed in our sample, findings reported here reflect the full sample, including identified outliers.

## Results

Results indicated that, regardless of diagnostic group, all adolescents performed above chance levels (*p*s <.05) on both the adult and child subtests of the FER task (see **Table 2**).

**Table 2:** Descriptive Statistics and One-Sample t-Tests for Facial Emotion Accuracy on the DANVA-2.

	DANVA-2 Subtest	Mean Accuracy (SD)	N	Comparison Value	95% CI for Mean Difference	*t*	df
ASD	Adult Faces	72.76% (11.08)	52	.25	44.67–50.84	31.07***	51
	Adult Happy Faces	89.74% (12.42)					
	Adult Sad Faces	78.85% (24.72)					
	Adult Angry Faces	59.29% (20.46)					
	Adult Fearful Faces	63.14% (20.96)					
	Child Faces	81.41% (12.66)	52	.25	52.89–59.94	32.128***	51
	Child Happy Faces	94.55% (11.30)					
	Child Sad Faces	89.10% (14.34)					
	Child Angry Faces	63.46% (29.34)					
	Child Fearful Faces	78.53% (17.57)					
Non-ASD	Adult Faces	75.10% (12.39)	40	.25	46.14–54.07	25.57***	39
	Adult Happy Faces	89.58% (14.95)					
	Adult Sad Faces	80.83% (18.32)					
	Adult Angry Faces	61.25% (20.81)					
	Adult Fearful Faces	68.75% (20.74)					
	Child Faces	83.54% (11.40)	40	.25	54.90–62.19	32.48***	39
	Child Happy Faces	95.83% (11.79)					
	Child Sad Faces	94.17% (9.66)					
	Child Angry Faces	61.67% (26.74)					
	Child Fearful Faces	82.50% (18.47)					

***p <.001. 95% CI for Mean Difference = 95% Confidence Interval for Mean Difference. As there are four face options for every trial, ¼ (or.25) is the comparison (chance) value against which response rates are compared. DANVA-2 Adult Face Accuracy, percentage of facial emotions correctly identified on the Diagnostic Analysis of Nonverbal Accuracy–Second Edition adult subtest; DANVA-2 Child Face Accuracy, percentage of facial emotions correctly identified on the Diagnostic Analysis of Nonverbal Accuracy–Second Edition child subtest.

**Figure 2:**
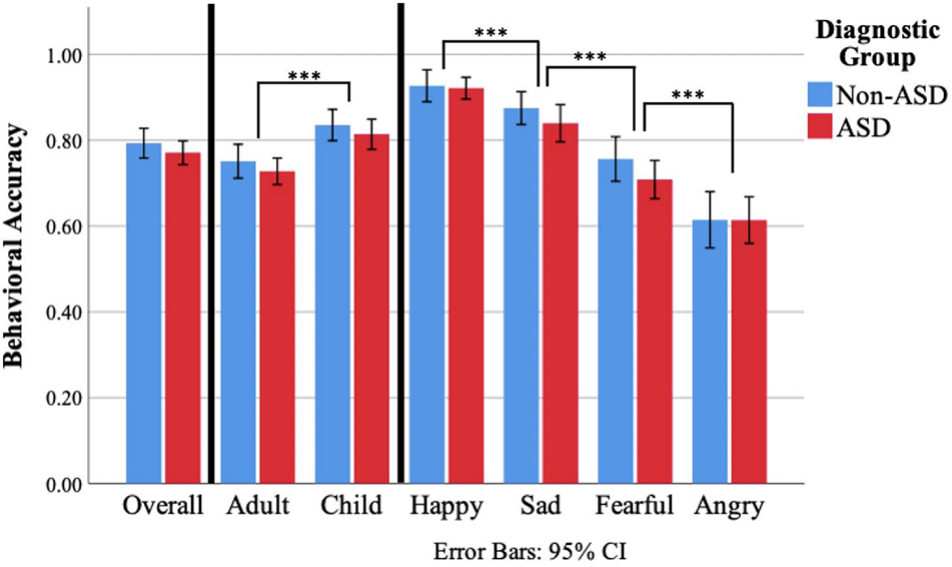
FER percent accuracy for the non-ASD and ASD diagnostic groups on the DANVA-2 by stimulus face age and emotion. Observed main effects of face age and emotion are denoted. A stepwise relationship was observed for comparisons between emotions such that accuracy for happy was significantly greater than sad, fearful, and angry; accuracy for sad was significantly greater than fearful and angry; and accuracy for angry was significantly greater than fearful, all at the *p* <.001 level. *** *p* <.001.

**Figure 3:**
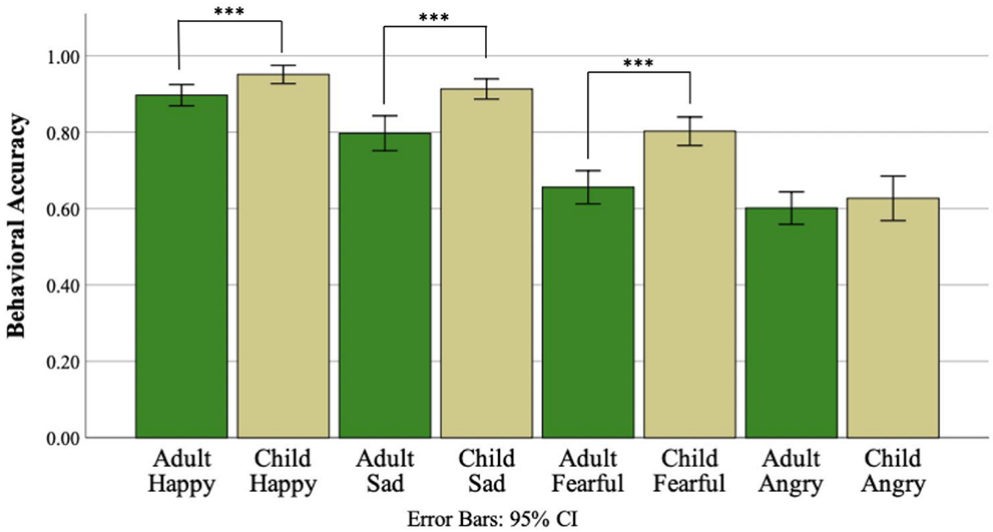
Percent FER accuracy for the adult and child subtests of the DANVA-2 by emotion. *** *p* <.001.

The three-way ANOVA testing for effects of face age, emotion, and diagnostic group indicated a main effect of face age (*F*(1,90) = 47.51, *p* <.001, $\eta_{p}^{2}=.35$, **Figure 2**), such that all participants were more accurate in identifying child compared to adult facial expressions of emotion. A main effect of emotion was also identified (*F*(3,270) = 95.80, *p* <.001, $\eta_{p}^{2}=.52$). *Post-hoc* pairwise comparisons using Bonferroni correction revealed an ordinal relationship of performance accuracy between the four emotions such that accuracy was greater for happy than sad (*p* <.001), sad than fearful (*p* <.001), and fearful than angry (*p* <.001). A main effect of diagnostic group was not identified (*F*(1,90) = 1.06, *p* =.31, $\eta_{p}^{2}=.01$). Similarly, there was no evidence of an interaction between face age and diagnostic group (*F*(1,90) =.01, *p* =.93, $\eta_{p}^{2}$ <.001) or emotion and diagnostic group (*F*(3,270) = 0.66, *p* =.58, $\eta_{p}^{2}$ =.007). However; an interaction between face age and emotion was observed (*F*(3,270) = 5.77, *p* =.001, $\eta_{p}^{2}$ =.06, **Figure 3**). *Post-hoc* paired samples t-tests indicated that participants demonstrated greater accuracy for child compared to adult faces for happy (*t*(91) = 3.61, *p* <.001), sad (*t*(91) = 4.98, *p* <.001), and fearful (*t*(91) = 6.46, *p* <.001), but not angry faces (*t*(91) =.84, *p* =.41). Evidence for a three-way interaction between face age, emotion, and diagnostic group was not observed (*F*(3,270) = 0.426, *p* =.73, $\eta_{p}^{2}$ =.005).

**Figure 4:**
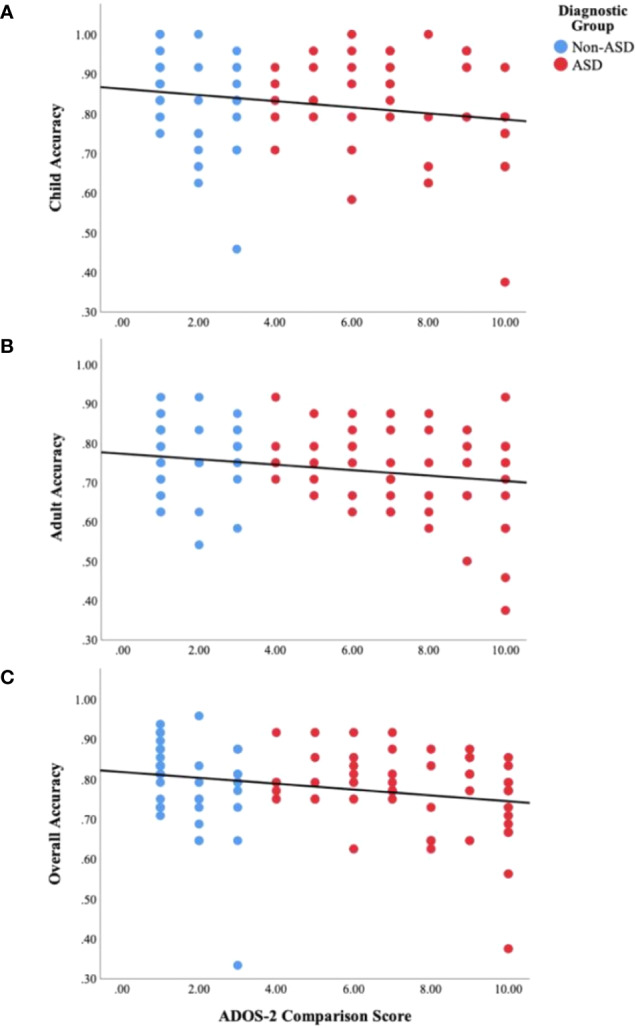
Scatter plots displaying the relationship between ASD symptom severity, as measured by the ADOS-2 Comparison Scores, and behavioral facial emotion recognition accuracy on the **(A)** child subtest (*r*(90) = −.21, *p* =.04), **(B)** adult subtest (*r*(90) = −.20, *p* =.06), and **(C)** overall task (*r*(90) = −.23, *p* =.03).

Results of Person's correlations for the combined sample indicated a significant relationship between ADOS-2 CS and total FER accuracy (*r*(90) = −.23, *p* =.03) as well as child accuracy (*r*(90) = −.21, *p* =.04), but only a marginal relationship with adult accuracy (*r*(90) = −.20, *p* =.06, see **Figure 4**). When examined separately by group, child accuracy (*r*(38) = −.34, *p* =.03) but not adult accuracy (*r*(38) = −.16, *p* =.31) or overall FER accuracy (*r*(38) = −.27, *p* =.09) was significantly associated with ADOS-2 CS for the non-ASD group. Conversely, for the ASD group, ADOS-2 CS was significantly associated with overall FER accuracy (*r*(50) = −.32, *p* =.02) and marginally associated with child accuracy (*r*(50) = −.27, *p* =.052) and adult accuracy (*r*(50) = −.27, *p* =.054).

Moderated regression models predicting ADOS-2 CS from adult and child accuracy indicated that the difference between FER performance on the adult and child subtest (e.g. strength of the OAB) did not predict ASD symptom severity for the non-ASD group (*B* = 4.59, *p* =.42), ASD group (*B* = −5.01, *p* =.72), or the two groups combined (*B* = −21.52, *p* =.16).

## Discussion

### General Discussion

The present study investigated whether a task of facial emotion recognition (FER) would elicit a performance advantage for own-age compared to other-age faces, known as an own-age bias (OAB) for individuals with and without ASD. In doing so, it was the first to explore whether the magnitude of an OAB is attenuated by ASD status or severity. Findings indicated that an OAB was elicited by the FER task for both adolescents with and without ASD and that the magnitude of the effect did not differ across groups. Similarly, overall accuracy differed ordinally by emotional expression such that recognition performance was best for happy, followed by sad, fearful, and angry. FER accuracy for individual expressions did not significantly differ by ASD group status. However, an interaction was observed between stimulus face age and emotion such that all participants demonstrated greater FER accuracy for child compared to adult faces for all emotions tested with the exception of anger. An examination of the relationship between FER accuracy and ASD symptom severity across the entire sample indicated a significant negative relationship between symptom severity and overall task accuracy as well as child accuracy, and a marginal negative relationship with adult accuracy. Follow-up analyses were all in the same direction, indicating that symptom severity was significantly associated with overall task accuracy and marginally associated with both child accuracy and adult accuracy for the ASD group. Symptom severity was significantly associated with poorer performance on only the child subtest for the non-ASD group. In line with the overall diagnostic status findings, results of a moderated regression approach indicated that the magnitude of the OAB was unrelated to ASD symptom severity in the individual groups as well as the combined sample.

In the present work, adolescents were observed to demonstrate greater performance accuracies for child compared to adult faces as hypothesized, regardless of ASD diagnostic status. This replicates previous findings that own-age biases are elicited by tasks of FER (10, 34), suggesting that increased contact with and exposure to peer faces during this developmental period, presumably through interactions with similarly-aged siblings, friends, and schoolmates, incurs a processing advantage for own-age faces (60). Alternatively, this processing advantage may not be due to an overall increase in visual experience with own-age faces, but rather increased social saliency and importance of peer interactions in adolescence (37, 61). Integrative accounts of the OAB, such as the categorization-individuation model (CIM; 62), posit that the processing advantage for own- compared to other-age faces reflects both an increase in perceptual expertise as well as a greater tendency to individuate, rather than categorize, faces of ingroup members. However, evidence in support of social-cognitive and integrative accounts of the OAB remains limited, with the majority of research findings substantiating experience-based accounts (11).

Interestingly, the presence of the OAB observed in this sample did not differ by ASD group. That is, adolescents with ASD demonstrated a robust performance advantage for child faces compared to adult faces that was no different than that of their peers without ASD. In conjunction with findings from the other-race effect (ORE) literature (44, 45), these results indicate that adolescents with ASD may be sensitive to both cumulative and more recent visual experience with faces.

Contrary to prediction, the diagnostic groups also did not differ on overall FER performance. Regardless of ASD status, adolescents demonstrated an ordinal relationship in their ability to accurately recognize specific emotional expressions such that performance was greatest for happy, followed by sad, fearful, and angry. This pattern of recognition accuracies replicates findings from the typical literature demonstrating that the ability to identify happy and sad facial expressions develops earlier than the ability to identify either fearful or angry facial expression (63). In addition to a main effect of expression type, an interaction between expression type and stimulus face age was also identified such that adolescents were better at identifying the expressions of child compared to adult faces for all emotions with the exception of anger. Interestingly, anger was also the expression that adolescents had the most difficulty in accurately identifying. Therefore, it may be the case that a processing advantage for own compared to other-aged faces may only become evident once a threshold level of expression specific accuracy is reached. Alternatively, this may be an artifact of the specific FER task, the DANVA-2, used in the present study.

In addition to examining differences in OAB and FER by diagnostic status, the present study also examined the impact of a continuous measure of ASD symptom severity. When diagnostic groups were combined, ASD symptom severity was found to be negatively associated with performance across the entirety of the task and the child subtest as well as marginally associated with poorer performance on the adult subtest. Although FER ability was not found to differ by diagnostic status, follow-up, group-specific correlational analyses indicated that the observed relationships between ASD symptom severity and overall performance as well as performance on the adult subtest may have been primarily driven by the ASD group. That is, ASD symptom severity was found to be significantly associated with overall task performance and marginally associated with performance on the adult subtest for individuals in the ASD group but not the non-ASD group. However, ASD symptom severity was found to be either significantly or marginally related to poorer performance on the child subtest for both individuals in the non-ASD and ASD diagnostic groups respectively. This suggests that the observed relationship between ASD symptom severity and performance on the child subtest was not primarily driven by adolescents that met diagnostic criteria for ASD. Overall, these findings replicate that of the existing literature which has identified broad deficits in FER for individuals with ASD (1–3). However, it should be noted that in the present sample impaired FER performance was only found when treating ASD symptom severity continuously, not categorically. This provides support for the argument that FER deficits associated with ASD may vary more subtly along dimensions of severity rather than being categorically distinct (48–50). It is also likely the case that measuring ASD symptom severity continuously afforded more power to detect differences in FER.

While ASD symptom severity was associated with overall FER performance, it did not predict the magnitude of the OAB (e.g. the discrepancy between performance on the child and adult subtests). This suggests that while ASD symptom severity may impact overall ability to recognize facial expressions it may not be due to an inability to incorporate more recent visual experiences into perceptual judgments. Many models of face-relevant perceptual expertise posit that exposure to exemplars of a face category results in the formation of a prototypic mental representation of that category. As exposure to a face category increases, its prototypic representation becomes more refined and more accurately reflects the distinguishing features of that category (9, 25). While these models were developed in the context of facial identity recognition tasks, it is likely the case that emotion identification also requires individuals to abstract and store prototypical representations of the basic emotions that can then be generalized across individuals.

Studies examining the development of prototype formation suggest that individuals with ASD demonstrate difficulty in abstracting prototypic representations of natural categories such as faces (27, 64, 65). If the OAB is a reflection of more refined prototypic representations for own- compared to other-age faces, then deficits in prototype formation should preclude individuals from demonstrating the effect. Findings here suggest that individuals with ASD may indeed be sensitive to increases in visual experience with own-age faces. However, given the relatively high performance accuracies observed for this task, it is possible that the impact of underlying deficits in prototype formation may only become apparent during more difficult tasks of FER (5, 18).

### Limitations and Future Directions

This work is the first to explore the impact of ASD status and symptom severity on the magnitude of an OAB. As such, there are notable limitations. First, the present study did not include an adult comparison group. Although a robust OAB was observed, without an adult comparison group, the possibility that this effect was due to systematic differences between the adult and child subtests cannot be definitively eliminated. Potential task specific factors include an imbalance in the difficulty of the adult- and child-subtests, differences in perceptual features between images in each subtest (e.g. luminance, contrast, and background), and the failure to randomize presentation order of adult and child faces. However, given the extensive literature demonstrating the reliability of this bias (11, 12), we can be relatively confident that this was not, in fact, the case. The lack of an adult comparison group may have also limited our ability to identify differences in the magnitude of an OAB associated with ASD. While deficits in FER are associated with ASD across the lifespan, evidence suggests that the greatest levels of impaired performance are observed in adulthood (5, 22, 29, 30). Thus, while ASD status and symptom severity appear unrelated to the strength of an OAB for adolescents, this may not hold at older ages, when deficits in FER are more pronounced.

Second, while the stimuli in the child-face subtest overlapped our participants in age, they were not perfectly age-matched. Given that our oldest adolescents were still in middle school, it is reasonable to presume they likely encountered children of younger ages during their typical school days and through engagement in extracurricular activities or time spent with siblings. However, this presumption of increased experience with peer faces may be less true of adolescents with ASD whom may experience greater ostracization from peers (66) and/or demonstrate less selective attention to peer faces in their daily environment (67, 68). Future work should take care to match ages of participants to stimuli more precisely as well as include a direct measure of visual facial experience. Ideally, future work will also incorporate experimental training-based designs into study methodologies to allow for direct manipulations of experience with faces of a target age as well as take into account additional characteristics of the sample that may impact task performance such as overall cognitive ability and adaptive functioning.

### Conclusions

Findings indicate the presence of an OAB, reflected in greater FER performance for child compared to adult faces, for adolescents, regardless of ASD diagnostic status or symptom severity. Although overall FER performance was poorer as a function of ASD symptom severity, the strength of the OAB was not influenced by either ASD categorical group membership or symptom severity. This suggests that recent visual experiences may not differentially influence the face processing abilities of adolescents with ASD and their typically developing peers. This work highlights the importance of leveraging theoretical perspectives established in the extant face processing literature on typically developing individuals in order to better understand the mechanisms underlying face processing deficits associated with ASD.

## Data Availability Statement

The datasets generated for this study will not be made publicly available but portions of the data may be accessed through the National Institute of Mental Health Data Archive here: https://nda.nih.gov/edit_collection.html?id=2421.

## Ethics Statement

The studies involving human participants were reviewed and approved by Stony Brook University Institutional Review Board. Written informed consent to participate in this study was provided by the participants' legal guardian/next of kin.

## Author Contributions

KH, PF, CK, and ML contributed to the conceptualization and refining of research ideas. KH, CK, and ML contributed to the acquisition of the data. KH and ML contributed to the analysis and interpretations of data. KH, PF, CK, and ML contributed to the drafting and revising of the manuscript for important intellectual content. KH, CF, and ML contributed to the creation of tables and figures. ML contributed to the creation of the research design and selection of measures. All authors contributed to manuscript revision, read and approved the submitted version.

## Funding

This research is funded by National Institute of Mental Health grant 1R01MH110585, awarded to ML (PI).

## Conflict of Interest

The authors declare that the research was conducted in the absence of any commercial or financial relationships that could be construed as a potential conflict of interest.
